# Molecular Mechanism of Ochratoxin A Transport in the Kidney

**DOI:** 10.3390/toxins2061381

**Published:** 2010-06-09

**Authors:** Naohiko Anzai, Promsuk Jutabha, Hitoshi Endou

**Affiliations:** 1 Department of Pharmacology and Toxicology, Kyorin University School of Medicine, 6-20-2, Shinkawa, Mitaka-shi, Tokyo 181-8611, Japan; Email: promsuk@ks.kyorin-u.ac.jp (P.J.); endouh@ks.kyorin-u.ac.jp (H.E.); 2 J-Pharma Co. Ltd., 2-16-8, Shinjuku, Shinjuku-ku, Tokyo 160-0022, Japan

**Keywords:** ochratoxin A, mycotoxin, organic anion transporter, kidney, nephrotoxicity

## Abstract

The mycotoxin, ochratoxin A (OTA), is thought to be responsible for Balkan endemic nephropathy. OTA accumulates in several tissues, especially in the kidneys and liver. The excretion of OTA into urine is thought to be mainly by tubular secretion, presumably via the organic anion transport system. Recently, several families of multispecific organic anion transporters have been identified: organic anion transporters (OATs), organic anion-transporting polypeptides (OATPs), oligopeptide transporters (PEPTs), and ATP-binding cassette (ABC) transporters, such as MRP2 and BCRP. These renal transporters mediate the transmembrane transport of OTA and play a pivotal role in the development of OTA-induced nephrotoxicity.

## 1. Introduction

Ochratoxin A (OTA), a structural analog of phenylalanine that contains a chlorinated dihydroisocoumarin moiety [1], is a mycotoxin produced as a secondary metabolite in different species of *Aspergillus ochraceus* and *Penicillium verrucosum* [2]. OTA may be distributed extensively in agricultural commodities and in the natural environment [3]. Contamination of foods with OTA, particularly of cereals, nuts, and coffee beans, has been noted in Eastern and Central Europe, North Africa, North America, and Japan [4]. Recently, OTA has received considerable attention because of its deleterious effects on human and animal health [5,6,7]. OTA accumulates in several tissues in the body, with the kidney being its main target, where it exerts toxic and carcinogenic effects [8,9]. Three forms of human renal disease, Balkan endemic nephropathy (BEN), chronic interstitial nephritis, and karyomegalic interstitial nephritis, appear to be caused, at least in part, by enhanced exposure to OTA [10]. However, the pathophysiological role of OTA in human renal disease has not yet been completely determined.

In the kidney, OTA mainly impairs proximal tubular functions and causes glucosuria, enzymuria, and a decrease in the transport of *para*-aminohippuric acid (PAH), a prototypical renal organic anion [11,12,13]. An *in vivo* study revealed that probenecid, a typical inhibitor of organic anion transporters (OATs), inhibited the renal clearance of OTA [14]. In addition, glomerular filtration of OTA is considered to be minimal, since more than 99% of OTA is bound to plasma proteins [15,16]. From these results, it is thought that the excretion of OTA into the urine is mainly by tubular secretion, presumably via the OAT system, and that this may play an important role in OTA accumulation and in the development of nephrotoxicity. Thus, knowledge of the fate of OTA that has reached the tubular lumen for its elimination is of crucial importance to understanding how OTA accumulates in renal tubular cells, and for developing possible strategies for prevention of OTA-induced nephrotoxicity.

In this review, we first summarize the findings from classical transport studies of OTA. Second, we briefly introduce the molecular identification of renal organic anion transporters. Third, we describe, by cloned OATs, the OTA transport properties. Through the text, we discuss the current idea implicating the function of the renal organic anion transporters as a determinant of OTA-induced nephrotoxicity. For further information concerning the roles of OTA as a signal modulator in the kidney, please refer to a precise review [17].

## 2. OTA Transport Systems in Classical Studies

Before the molecular identification of renal OATs, there were several lines of evidence that showed transcellular transport of OTA occurred via carriers (transporters). 

### 2.1. Renal Slice Study

In 1979, Berndt and Hayes [18] found that daily administration of OTA in rats caused altered renal slice transport of several organic compounds. In addition, there was persistent urinary hypoosmolality coupled with excessive glucose and protein excretion. They also examined the direct addition of OTA to fresh renal cortex slices and observed depressed transport of some organic compounds. Based on these findings, they concluded that OTA has the capacity to produce alterations in renal function that are suggestive of nephrotoxicity.

Friis *et al.* [19] also found, by using pig renal cortical slices, that OTA enters proximal tubular cells by the common organic anion transport system for PAH and phenolsulphophthalein (PSP).

### 2.2. Membrane Vesicle Study

In 1988, Sokol *et al.* [20] examined that the effect of OTA on the transport of [^3^H]PAH in brush border membrane vesicles (BBMV) and in basolateral membrane vesicles (BLMV) from canine kidneys. OTA was as effective an inhibitor of PAH uptake, in both membranes, as probenecid. Based on the finding that OTA produced *trans*-stimulation of PAH transport in both BBMV and BLMV, they concluded that OTA is transported across both of these membranes via the renal organic anion transport system. 

### 2.3. Micropuncture Study

In 1997, Zingerle *et al.* [21] micropunctured *in situ* the superficial early proximal and early distal tubules of male Wistar rats and showed, after microinfusion of OTA into superficial nephrons, that OTA was reabsorbed in the proximal as well as the distal parts of the nephron. They also reported that: (1) one-third of reabsorption takes place in the distal tubule or the collecting duct, or both, and two-thirds take place in the proximal tubule; (2) "distal" reabsorption can be explained, at least partially, by nonionic diffusion; (3) "proximal" reabsorption was partially mediated by the H^+^-dipeptide cotransporter and (4) reabsorption of filtered and secreted OTA delay its excretion and may lead to accumulation of the toxin in the renal tissue. 

### 2.4. Tubular Suspension Study

Groves’s group [22] examined the transport of OTA across the renal peritubular membrane in suspensions of rabbit renal proximal tubules. OTA transport across the peritubular membrane was a high-affinity, low-capacity carrier-mediated process with a K_m_ value of 1.4 µM. The apparent K_i_ value for [^3^H]PAH uptake by OTA inhibition was 1.5 µM, which is similar to the K_m_ value for OTA uptake in tubule suspensions, and suggests that OTA could be a substrate for the organic anion pathway. The capacity and affinity for peritubular OTA transport were 40-fold lower and more than 100-fold greater, respectively, than those measured for the peritubular uptake of [^3^H]PAH in tubule suspensions. Probenecid-sensitive, PAH-insensitive uptake of OTA suggested that at least one mediated pathway, other than the organic anion transporter, was involved in the peritubular uptake of OTA. The ability of octanoic acid to inhibit OTA transport to the same extent as probenecid, and to a greater extent than PAH, suggests that a separate fatty acid transport pathway may be involved in the accumulation of OTA in suspensions of rabbit renal proximal tubules.

### 2.5. Isolated Tubular Study

Groves *et al.* [23] studied the transepithelial transport of OTA using primary cultures of rabbit renal proximal tubule cells grown under improved culture conditions. The basal-to-apical (B-to-A) transepithelial flux, *i.e.*, secretion, of this fluorescent organic acid was measured in the primary cultures of rabbit renal proximal tubule cells. The B-to-A flux of OTA increased with time and reached a steady state after 12 h. On the other hand, the A-to-B flux, *i.e.*, reabsorption, of OTA was minimal over time. The secretory flux of OTA was as much as eight-fold greater than the reabsorptive flux, indicating that a net secretion is the primary mechanism for OTA clearance by the proximal tubule. The high affinity measured for the B-to-A flux of OTA suggests that at concentrations typical of naturally occurring exposures, transepithelial secretion by the organic anion transport pathway represents a significant avenue for excretion of this mycotoxin by the renal proximal tubule.

### 2.6. Cell Monolayer Study

Schwerdt *et al.* [24] investigated the transepithelial transport of OTA across monolayers of two collecting duct-derived cell clones (Madin-Darby canine kidney cells (MDCK)-C7 and MDCK-C11 cells, resembling principal and intercalated cells, respectively), either from the apical to the basolateral side, or *vice versa*, on permeable supports. They showed that OTA is transported across the apical membrane of MDCK cells by both non-ionic diffusion and by an H^+^-dipeptide cotransporter, and concluded that the reabsorption of OTA in the collecting duct contributes to the observed long half-life of OTA in the mammalian body. 

Then, Schwerdt *et al.* [25] studied, in detail, the characteristics of apical uptake of [^3^H]OTA in MDCK-C11 cells. They showed that apical uptake of OTA occurs by a Na^+^-independent transport. The uptake is mediated partly by H^+^-dipeptide cotransport (30%), partly by organic anion transporter (20%), and partly by diffusion (20%). The remaining part occurs by as-yet-unidentified, but pH-dependent transport mechanisms. Acidic urine in distal parts of the nephron thus provides the main risk for the OTA uptake that leads to its reabsorption. Thus, alkalinization of the urine should help prevent this reabsorption. Bahnemann *et al.* [26] investigated the kinetics of secretion and the extent of urine and tissue accumulation *in situ* by using a non-filtering amphibian kidney model. They demonstrated that transepithelial secretion is an effective way of accumulating OTA in the tubular lumen, and thus, of promoting OTA’s urinary excretion. Basolateral transport, via the organic anion carrier and an amino acid carrier, involves the active steps of transepithelial secretion. Luminal exit of OTA is a passive process. Furthermore, tissue accumulation by basolateral active transport supports the toxic action of OTA on proximal tubular cells. 

Next, Sauvant *et al.* [27] investigated the effect of OTA on proximal tubule-derived opossum kidney (OK) cells, because a decline in the net secretion of PAH was observed after chronic exposure to OTA *in vivo*. They found that long-term incubation with free OTA in the nanomolar range reduces the activity of the organic anion transporter without influencing general cell function, and that OTA seems to act preferentially on organic anion transport, by affecting the exchange of organic anions and dicarboxylates. This effect leads to the reduction of its own secretion.

Welborn *et al.* [28] studied the peritubular transport of fluorescent OTA into single proximal tubule segments of the rabbit by using epifluorescence microscopy. Initial rates of OTA uptake into the S2 segments were saturable and adequately described by Michaelis-Menten kinetics (K_m_: 2.2 µM). Several lines of evidence indicated that peritubular uptake of OTA in S2 segments was effectively limited to the “classical” organic anion transporter. The high affinity and relatively high capacity of this pathway for OTA suggests that the peritubular uptake may be a significant avenue for the entry of this toxin into proximal tubule cells.

**Figure 1:**
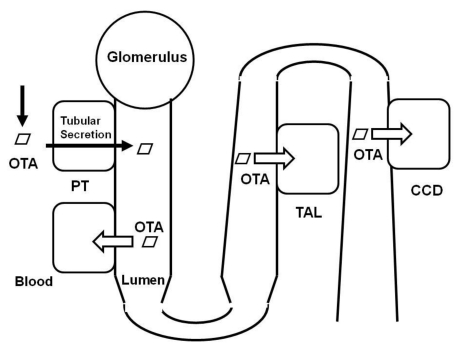
Transport systems for OTA along the nephron. OTA secretion is performed by the proximal tubular organic anion secretion pathway. OTA is reabsorbed along the nephron from apical side. Abbreviations, PT: proximal tubules, TAL: thick ascending limb of the loop of Henle, CD: collecting duct.

Figure 1 depicts the summary of the classical concept for OTA transport systems in the kidneys. Tubular secretion of OTA is mainly performed by the organic anion transport system in the proximal tubules and secreted OTA was reabsorbed in the proximal tubules, distal tubules (TAL), and the collecting ducts (CCD) by several transport systems, leading to the delay of OTA excretion and the accumulation of OTA in tubular cells.

## 3. Molecular Identities of Renal Organic Anion Transporters

**Figure 2:**
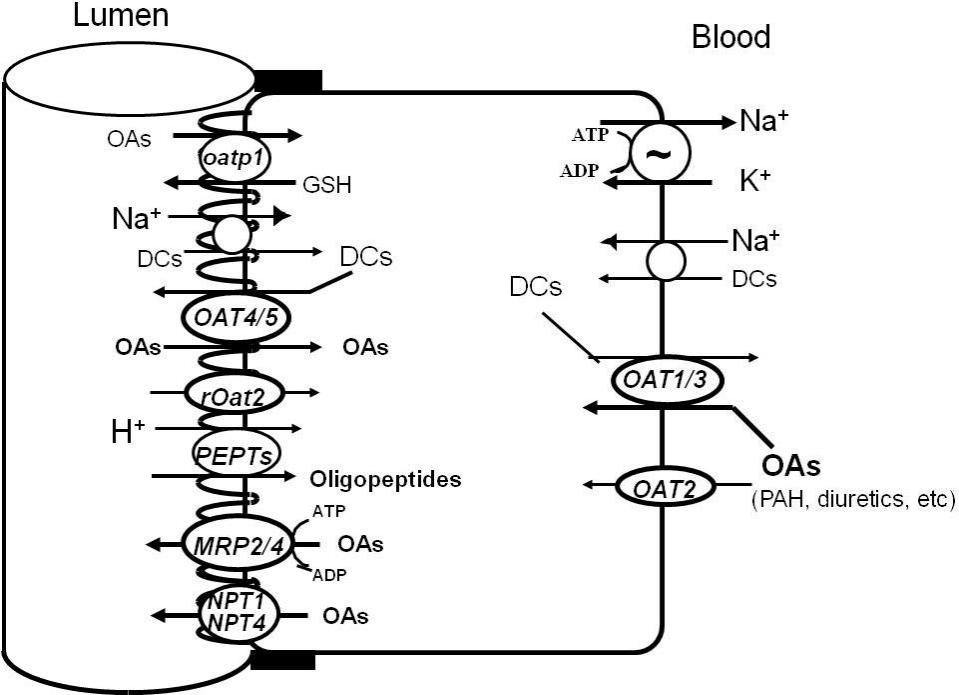
Proposed model of organic anion transporters in renal proximal tubules. Abbreviations, OAs: organic anions, DCs: dicarboxylates, MCs: monocarboxylates, GSH: glutathione.

In this section, we will briefly introduce the renal transporters for organic anionic drugs. A number of reviews [29,30,31,32,33,34] on organic anion transporters are available. Figure 2 shows cloned organic anion transporters of renal proximal tubules that have been reported to date.

### 3.1. Organic Anion Transporter (OAT) Family SLC22

**OAT1:** Rat Oat1 mRNA is expressed predominantly in the kidneys and weakly in the brain [35]. In the kidneys, OAT1 protein is localized in the basolateral membrane of proximal tubular cells. OAT1-mediated uptake of PAH is stimulated by an outwardly directed concentration gradient of dicarboxylates, such as α-ketoglutarate. The substrate selectivity of OAT1 is markedly broad. These substrates include endogenous substances, such as dicarboxylates, cyclic nucleotides, and prostaglandins, and exogenous substances, such as various anionic drugs and environmental compounds [36].

**OAT2:** Oat2 was originally isolated from the rat liver as a novel liver-specific transport protein with an unknown function [37]. OAT2 is expressed predominantly in the liver and weakly in the kidneys. The typical substrates of OAT2 are salicylate, acetylsalicylate, prostaglandin E_2_ (PGE_2_), dicarboxylates, PAH, zidovudine (AZT), and tetracycline [31].

**OAT3:** Oat3 was initially isolated from the rat brain [38]. OAT3 mRNA is expressed in the kidneys and the brain. In the kidneys, OAT3 is localized in the basolateral membrane of proximal tubular cells. Like OAT1, OAT3 recognizes a broad spectrum of substrates. It mediates the high affinity transport of PAH, estrone sulfate (ES), OTA, and various drugs, including the cationic drug cimetidine, in exchange for dicarboxylates inside cells [31].

**OAT4:** OAT4 was cloned from human kidneys [39]. OAT4 mRNA is expressed in the kidneys and is localized in the apical membrane of proximal tubular cells. When expressed in *Xenopus* oocytes, OAT4 mediates the Na^+^-independent, high-affinity transport of ES, dehydroepiandrosterone sulfate, OTA, PGE_2_, and PGF_2_α [31].

**Oat5:** Oat5 was identified from the rat kidney [40]. It is expressed exclusively in the kidneys. Rat Oat5 is localized in the apical side of proximal tubules. Oat5 mediates the transport of steroid sulfates, as well as OTA.

**Oat8:** Oat8 was recently identified from the rat kidney [41]. It is expressed exclusively in the kidneys. Rat Oat8 protein is localized in the collecting duct. Additionally, it is colocalized with V-ATPase in plasma membranes and the intracellular vesicles of various subtypes of intercalated cells. Like Oat5, Oat8 mediates the transport of steroid sulfates, as well as OTA.

**Oat9:** Oat9 has recently been identified from the mouse kidney [42]. It is expressed in the kidneys and in the liver. Oat9 has two isoforms: a long form and a short form. The short form of Oat9 (Oat9S) is functional and mediates the transport of zwitterion L-carnitine, as well as cimetidine and salicylic acid. Oat9 is localized in the apical side of proximal tubules.

**OAT10:** Bahn *et al.* showed that hORCTL3 (human organic cation transporter like 3; OCTL1), an orphan transporter expressed mainly in the kidneys, transports nicotinate, PAH, and uric acid. They renamed it OAT10 [43]. They suggested that OAT10 is the molecule responsible for cyclosporine A-induced hyperuricemia.

### 3.2. Organic Anion Transporting Polypeptide (OATP) Family SLC21/SLCO

The first member of this family, oatp1, was identified from the rat liver, by an expression cloning method, as a sodium-independent bile acid transporter [44]. Thus far, 11 human isoforms and 14 rat isoforms have been identified in the OATP family [45]. Although some OATPs are selectively involved in the hepatic uptake of bulky and relatively hydrophobic organic anions, most OATPs are expressed in many tissues, such as the blood-brain barrier, choroid plexus, lungs, heart, intestine, kidneys, placenta, and testes. The OATP family is divided into six sub-families (OATP1–OATP6). There are considerable species differences in the OATP families among rodents and humans. Among human OATPs, only OATP4C1 is mainly expressed in the kidneys [46]. Oatp1a3v1 (previous name: Oat-k1) and Oatp1a3v2 (previous name: Oat-k2) are specifically expressed in the rat. Oatp1a1 (previous name: oatp1), Oatp1a5 (previous name: oatp3), Oatp1a6 (previous name: oatp5), and Oatp4c1 are expressed in rodent kidneys. Orthologs of these isoforms, except of OATP4C1, are absent in humans. Because of the above-mentioned remarkable species differences in OATP, it is difficult to assign distinct physiological roles to each OATP in the kidneys. Several important substances, which are mainly excreted via the kidneys, are preferable substrates for the OATP family. Recently, OATP4C1 has been revealed to be a digoxin transporter [46]. OATP4C1 is expressed exclusively in the basolateral membrane of proximal tubular cells and mediates the high-affinity transport of digoxin (K_m_: 7.8 µM) and ouabain (K_m_: 0.38 µM), as well as thyroid hormones, such as triiodothyronine (K_m_: 5.9 µM). 

### 3.3. Type I Sodium/Phosphate Transporter (NPT) Family SLC17

Molecular studies have revealed that type I phosphate transporters (SLC17), a family of proteins initially characterized as phosphate carriers, are expressed at the apical membrane of renal proximal tubular cells. They mediate the transport of organic anions [47]. Mouse and human NPT1 were shown to mediate the transport of various organic anions in a chloride-dependent manner. Moreover, because human NPT1 exhibits an affinity for PAH, which corresponds to the results of previous studies using brush-border membrane vesicles, NPT1 has also been suggested to represent a classical voltage-dependent PAH transporter. However, the influence of membrane potential on PAH transport was not demonstrated [48]. 

Recently, we have found that the SLC17 family member, NPT4, mediates the transport of PAH in a voltage-sensitive manner, indicating that NPT4 may be involved in the apical drug efflux pathway [49].

### 3.4. Oligopeptide Transporter (PEPT) Family SLC15

Peptide transporters are involved in the electrogenic, H^+^-dependent transport of small peptides. They are also involved in the transport of various peptide-like drugs, such as β-lactam antibiotics, angiotensin-converting enzyme (ACE) inhibitors, and anticancer drugs [50]. Two peptide transporters, designated as PEPT1 and PEPT2, have been cloned. PEPT1, a low-affinity/high-capacity transporter, was first cloned from the rabbit intestine, and subsequently, from the rat and human intestines. Rat Pept1 is localized to the apical side of intestinal epithelial cells and in early regions (S1 segments) of the apical proximal tubules. PEPT2, a high-affinity/low-capacity transporter, appears to have a different tissue localization than that of PEPT1. PEPT2 is highly expressed in the kidney, but not in the intestine. Rat Pept2 is localized to the apical side of the proximal tubule in more distal regions (S3 segments).

### 3.5. Multidrug Resistance-Associated Protein (MRP) Family ABCC

The MRP family consists of primarily active transporters with ATP-binding cassette motifs. The prototype of this family is P-gp [51], which extrudes various hydrophobic molecules. P-gp particularly extrudes antineoplastic compounds, such as vincristine, vinblastine, adriamycin, and daunorubicin. P-gp confers multidrug resistance upon cancer cells [52]. MRP1 and MRP2 were isolated from multidrug resistant cancer cells that did not express P-gp. In addition to antineoplastic drugs, MRP2 transports glucronides and cysteine conjugates. MRP2 is expressed in the canalicular membrane of hepatocytes [53]. To date, many isoforms have been identified in the MRP family. Several of these isoforms are expressed in the apical membrane of proximal tubular cells. MRP members in proximal tubular cells supposedly function as extrusion pumps for organic anions, especially for large and hydrophobic organic anions, from the apical membrane. Regarding the family’s renal physiology and pharmacology, particular attention should be paid to two isoforms, namely, MRP2 and MRP4. MRP2 has been shown to transport PAH, but its affinity for PAH is low (K_m_: 2 mM). In contrast, human MRP4, which is also localized in the apical membrane of proximal tubular cells, transports PAH with a much higher affinity (K_m_: 160 µM) than that of MRP2. Furthermore, real-time PCR and Western blot analysis have shown that the renal cortical expression of MRP4 is approximately five-fold higher than that of MRP2 [54]. These data demonstrate that MRP4 plays a certain role in the efflux of PAH and several small hydrophilic organic anions, such as urate, cAMP, and cGMP, into the tubular lumen [55].

### 3.6. Breast Caner Resistance-Associated Protein (BCRP) ABCG2

BCRP, a member of the ABC transporter family to which MRP4 belongs, was recently reported to transport uric acid [56]. In addition, the finding that the Q141K polymorphism associated with gout leads to decreased urate excretion capacity suggests that ABCG2 contributes to urate excretion at the apical membrane of renal proximal tubules or at the luminal membrane of the intestine [56], or both.

## 4. OTA Transport Properties in Cloned Organic Anion Transporters

### 4.1. Basolateral OATs

Tsuda *et al.* [57] investigated the transport of OTA by rat kidney-specific organic anion transporter 1 (rOat1). When expressed in *Xenopus* oocytes, rOat1 mediated the sodium-independent uptake of OTA (K_m_: 2.1 µM). Piroxicam, which has been shown to prevent the nephrotoxicity of OTA, inhibited rOat1-mediated uptake of OTA. The research group investigated the transport of OTA and OTA’s effect on cell proliferation and viability by using cells derived from the mouse kidney terminal proximal tubule (S3) that were transfected with rOat1 cDNA (S3 rOat1). S3 rOat1 mediated the saturable transport of OTA (K_m_: 0.57 µM). Proliferation of S3 rOat1 cells was suppressed when exposed to OTA (2 and 10 µM). This suppression was rescued by the addition of PAH (1 mM) to the media. These results indicated that OTA is transported by rOat1 and that the accumulation of OTA via rOat1 in proximal tubular cells is the primary event in the development of OTA-induced nephrotoxicity.

Jung *et al.* [58] then investigated the characteristics of OTA transport by multispecific human organic anion transporters (hOAT1 and hOAT3, respectively) using cells from the second segment of the proximal tubule (S2) from mice that stably expressed hOAT1 and hOAT3 (S2 hOAT1 and S2 hOAT3). S2 hOAT1 and S2 hOAT3 exhibited a time- and concentration-dependent uptake of [^3^H]OTA (K_m_: 0.42 µM for hOAT1, 0.75 µM for hOAT3). PAH, probenecid, piroxicam, octanoate, and citrinin inhibited [^3^H]OTA uptake by hOAT1 and hOAT3 in a competitive manner (K_i_: 4.29-3080 µM), with different orders of potency for hOAT1 and for hOAT3. These results indicate that hOAT1and hOAT3 both mediate the high-affinity transport of OTA on the basolateral side of the proximal tubule. Additionally, the results show that hOAT1-mediated and hOAT3-mediated OTA transport differs in how they are influenced by the substrates of OATs. These pharmacological characteristics of hOAT1 and hOAT3 may be significantly related to the development of OTA-induced nephrotoxicity in the human kidney.

Zhang *et al.* [59] compared the characteristics of several cloned rabbit organic ion transporters expressed in cultured cells with their behavior in intact rabbit renal proximal tubules (RPT) to determine the contribution of each to the basolateral uptake of OTA. The activity of organic anion transporters OAT1 and OAT3 proved to be distinguishable because OAT1 had a high affinity for PAH (K _t_ of 20 µM), and did not support estrone sulfate (ES) transport, whereas OAT3 had a high affinity for ES (K_t_ of 4.5 µM), and had a weak interaction with PAH (IC_50_ > 1 mM). In contrast, both transporters robustly accumulated OTA. Intact RPT also accumulated OTA, with OAT1 and OAT3 each being responsible for approximately 50%; ES and PAH each reduced uptake by approximately 50%, and the combination of the two eliminated mediated OTA uptake. They concluded that the fractional contribution of different organic ion transporters to renal secretion is influenced by their affinity for substrate and by their relative expression level in RPT.

### 4.2. Apical OATs

Babu *et al.*, using mouse proximal tubule cells stably transfected with OAT4 (S2 OAT4) [60], examined the characteristics of OTA transport by OAT4. Immunohistochemical analysis revealed that the OAT4 protein was localized to the apical side of the proximal tubule. S2 OAT4 exhibited a time- and concentration-dependent increase in OTA uptake (K_m_: 22.9 µM). OAT4-mediated OTA uptake was inhibited by several substrates for OATs. Probenecid, piroxicam, octanoate, and citrinin inhibited OTA uptake by OAT4 in a competitive manner (K_i_: 44.4–336.4 µM). The addition of OTA resulted in a slight decrease in the viability of S2 OAT4 when compared with the mock. These results indicate that hOAT4 mediates the high-affinity transport of OTA on the apical side of the proximal tubule. Considering the driving force of OAT4, as indicated by Ekaratanawong *et al.* [61], OAT4 seems to function as a reabsorption pathway for OTA at the apical membrane of renal proximal tubules.

**Figure 3:**
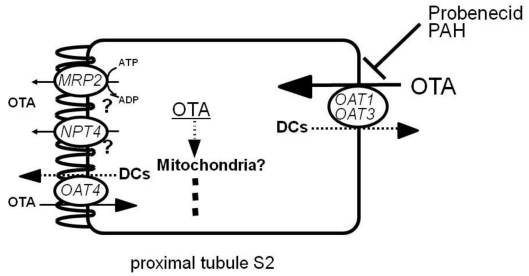
OTA secretion in proximal tubular cells in humans. Organic anion/dicarboxylates (DCs) exchangers OAT1 and OAT3 function as entrance pathways for OTA, and NPT4 and MRP2 may be its exit route in proximal tubular cells. OAT4 at the apical membrane may function as a reabsorptive pathway for OTA. When probenecid (inhibitor of OATs) or PAH (substrate for both OAT1 and OAT3) is coadministered with OTA, they inhibit the tubular secretion of OTA, thus reducing its potential risk.

Recently, we have identified an orphan transporter, NPT4 (*SLC17A3*), as a novel human voltage-driven organic anion transporter at the apical side of proximal tubules. We characterized its multispecific transport properties for organic anions, including PAH and several diuretics [49]. We also examined whether or not NPT4 mediates OTA transport by using *Xenopus* oocytes expressing hNPT4 [62]. hNPT4 demonstrated time- and concentration-dependent uptake of OTA. Like in the transport of PAH, OTA uptake by hNPT4 was enhanced in the medium with replacement of K^+^ by Na^+^. This suggests voltage-driven transport of OTA by hNPT4. hNPT4-mediated OTA uptake was inhibited by several organic anions, such as probenecid and piroxicam. These results indicate that hNPT4 is an apical efflux pathway for OTA entering via basolateral OAT1 or OAT3 (or both) in renal proximal tubules (Figure 3).

### 4.3. OATPs

Takeuchi *et al.* characterized the interactions of various compounds with kidney-specific organic anion transporters Oat-k1 and Oat-k2 [63]. By using MDCK cells stably transfected with Oat-k1 or Oat-k2 cDNA, the antitumor drug methotrexate, OTA, endogenous organic anions (thyroid hormones, taurocholic acid, and conjugated steroids), and the antiretroviral drug zidovudine were shown to be substrates for these transporters. These results suggested that Oat-k1 and Oat-k2 could serve as multispecific transporters, mediating the transport of a wide variety of endogenous substances, xenobiotics, and their metabolites in the kidney, presumably via several interaction sites in their molecules.

### 4.4. MRP2

The ATP-dependent membrane transporters, P-glycoprotein (P-gp), MRP2, and BCRP, are localized in the luminal membranes of the intestines and liver, as well as in the kidney. They counteract absorption and increase excretion of xenobiotics and drugs. Leier *et al.* investigated the transport function of renal apical multidrug resistance protein MRP2 (ABCC2) using PAH and OTA as transport substrates [64]. Membrane vesicles from HEK-MRP2 cells containing recombinant human MRP2 and from control vector-transfected HEK-Co cells were incubated with various concentrations of [^3^H]PAH and [^3^H]OTA. The net ATP-dependent transport into inside-out vesicles was determined (K_m_ for PAH: 880 µM). OTA inhibited MRP2-mediated PAH transport (IC_50_: 58 µM). OTA was itself a substrate for MRP2. 

Schrickx *et al.* [65] investigated the absorption and secretion of OTA in a Caco-2 cell model. Caco-2 cells secreted OTA to the luminal side in a concentration-dependent manner. This secretory permeability was higher than the absorptive permeability, while the absorptive permeability remained constant for all OTA concentrations tested. The secretion decreased and absorption increased in the presence of the MRP-inhibitor MK571, the P-gp and BCRP inhibitor GF120918, and the BCRP-inhibitor Ko143, suggesting that the secretion of OTA is mediated by MRP2 and BCRP. Cyclosporine A also decreased the secretory permeability, but did not affect absorptive permeability. PSC833 changed neither the absorption nor the secretion of OTA. Hence, it can be suggested that OTA is a substrate for MRP2 as well as for BCRP. These findings are of interest for evaluating mycotoxin absorption after oral ingestion, tissue distribution, and particularly, mycotoxin excretion pathways, including renal, biliary, and mammary gland excretion.

## 5. Transport of OTA in Extrarenal Tissues

### 5.1. Intestine

OTA is absorbed from the small intestine and binds to serum albumin in plasma. The prolonged half-life of OTA results from its reabsorption by the proximal tubules and via enterohepatic circulation. Berger *et al.* [66] investigated the mechanism by which OTA crosses the intestine. They used a cell culture system consisting of Caco-2 cells as an *in vitro* model of the human intestinal epithelium. Cytotoxicity assays on proliferating Caco-2 cells showed that OTA (0.4 µM) inhibits MTT reduction by 50%. Transepithelial transport and intracellular accumulation of OTA were studied in Caco-2 cells and were differentiated in bicameral inserts. At pH 7.4, OTA is transported preferentially in the basolateral (BL) to apical (A) direction, suggesting a net secretion. Conditions closer to the *in vivo* situation in the duodenum (A: pH 6.0, BL: pH 7.4) increased intracellular accumulation and transepithelial transport. A to BL transport becomes higher than BL to A transport, suggesting OTA absorption. Addition of serum albumin in the BL compartment further increases OTA absorption across Caco-2 cells and suggests that *in vivo* OTA transport from the serosal to the luminal side of enterocytes is prevented, due to its binding to plasma proteins. A and BL transport and intracellular accumulation of OTA are increased in the presence of non-specific inhibitors of MRPs (indomethacin, genistein, and probenecid) and in the presence of 1-chloro-2,4-dinitrobenzene (biotransformed into 2,4-dinitrophenyl-glutathione, a specific inhibitor of MRPs), but are not affected by verapamil, an inhibitor of P-gp. This suggests that MRP2 might be involved in transepithelial transport. Therefore, absorption of OTA across the intestinal mucosa would be limited, due to its excretion through MRP2 at the apical pole of enterocytes.

In addition, as mentioned in section *3.5*, ABC transporters, such as P-gp, MRP2, and BCRP, which are localized in the luminal membranes of the intestine, counteract absorption and increase excretion of xenobiotics and drugs. Schrickx *et al.* [65] suggested that OTA is a substrate for MRP2 as well as for BCRP.

### 5.2. Liver

Upon intestinal absorption, a large part of OTA is taken up by hepatocytes and eliminated into bile. Kontaxi *et al.* [67] characterized hepatocellular uptake of [^3^H]OTA in isolated rat hepatocytes. A saturable (K_m_: 18.9 µM) and energy-dependent OTA transport was found. This uptake was inhibited by various bile acids, sulfobromophthalein (BSP), and the thrombin inhibitor CRC 220. Because all inhibitors are substrates of the organic anion-transporting polypeptide (oatp), a hepatic carrier [44], uptake experiments were performed in oatp cRNA-injected *Xenopus* oocytes. These studies revealed an oatp-specific OTA uptake (K_m_: 16.6 µM). Known oatp substrates *cis*-inhibited OTA uptake in oatp cRNA-injected oocytes in close correlation with the results obtained from isolated hepatocytes. These results reveal OTA as a substrate for oatp. They further support the multispecific nature of oatp-mediated transport and stress the importance of this carrier for hepatic clearance of xenobiotics.

As mentioned before, Schrickx *et al.* [65] suggested that OTA is a substrate for MRP2 and BCRP, which are localized in the canalicular membranes of the liver.

### 5.3. Blood-brain Barrier

Ose *et al.* [68] investigated the role of a multispecific organic anion transporter, Oatp1a4/Slco1a4, in drug transport across the blood-brain barrier. *In vitro* transport studies using HEK293 cells expressing mouse Oatp1a4 revealed the following compounds as Oatp1a4 substrates: pitavastatin (K_m_: 8.3 µM), rosuvastatin (K_m_: 12 µM), pravastatin, taurocholate (K_m_: 40 µM), digoxin, and OTA. Double immunohistochemical staining of Oatp1a4 with P-gp or glial fibrillary acidic protein demonstrated that Oatp1a4 signals partly colocalized with P-gp signals, but not with glial fibrillary acidic protein, suggesting that Oatp1a4 is expressed in both the luminal and the abluminal membranes of mouse brain capillary endothelial cells. The brain-to-blood transport of pitavastatin, rosuvastatin, pravastatin, and taurocholate after microinjection into the cerebral cortex was significantly decreased in Oatp1a4(−/−) mice, compared with that in wild-type mice. The blood-to-brain transport of pitavastatin, rosuvastatin, taurocholate, and OTA, determined by *in situ* brain perfusion, was significantly lower in Oatp1a4(−/−) mice than in wild-type mice.

## 6. OTA as a Regulator of Transporter Expression

In rodents, OTA intoxication impairs various proximal tubule functions, including secretion of PAH, possibly via affecting the renal organic anion transporters (Oat). However, an effect of OTA on the activity/expression of specific Oats in the mammalian kidney has not been reported. Zlender *et al.* studied tubular integrity by microscopy, abundance of basolateral (rOat1, rOat3) and brush-border (rOat2, rOat5) rOat proteins by immunochemical methods, and expression of rOats mRNA by RT-PCR in kidneys from male rats gavaged with various doses of OTA every second day for 10 days [69]. The OTA treatment caused (1) dose-dependent damage of the cells in the S3 segments of medullary rays; (2) a dual effect upon rOats in the proximal tubules with low doses upregulating the abundance of all rOats and high doses downregulating the abundance of rOat1, and (3) unchanged mRNA expression for all rOats at low OTA doses, and downregulation of rOats at high OTA doses. Changes in the expression of renal Oats were associated with enhanced OTA accumulation in tissue and excretion in urine, whereas the indicators of oxidative stress either remained unchanged (malondialdehyde, glutathione, 8-hydroxydeoxyguanosine) or became deranged (microtubules). While OTA accumulation and downregulation of rOats in the kidney are consistent with the previously reported impaired renal PAH secretion in rodents intoxicated with high OTA doses, the post-transcriptional upregulation of Oats at low OTA doses may contribute to OTA accumulation and development of nephrotoxicity.

## 7. Conclusion

Renal organic anion transporters function as an entrance pathway for several xenobiotics and contribute to their accumulation in proximal tubular cells. In this review, we showed that nephrotoxic compounds, such as OTA, are substrates for renal drug transporters by transporter-stably expressing cell lines [57,58,60]. Use of transporter-stably expressing cells (S2 OATs), together with non-expressing cells (S2 mock), is helpful for checking the possibility of transporter-mediated nephrotoxicity. Thus, transporter-stably expressing cell lines seem to be useful systems as an animal alternative model for the screening of potential nephrotoxic compounds.

Besides OTA, the nephrotoxic effects of carbapenem antibiotics and antiviral drugs, such as adefovir and cidofovir, are closely associated with OATs [70]. The nephrotoxicity of these compounds could be reduced by the coadministration of other substrates of OATs or of inhibitors of OATs. Indeed, recently, a new application of probenecid as a nephroprotectant in therapy with the antiviral drug cidofovir was determined [71]. When probenecid is coadministered with cidofovir, probenecid inhibits the tubular accumulation of cidofovir, leading to the reduction of its potential risk. Thus, blockade of transport by competitors eliminates or reduces the nephrotoxic response.

An understanding of the molecular mechanism of renal organic transport is essential to achieving desired therapeutic outcomes in response to chemical exposure and drug interactions, to understanding the progression of some disease states, and to predicting the influence of genetic variation upon these processes.

Renal tubular secretion and reabsorption of OTA, presumably via the OAT system, may play an important role in OTA accumulation and development of nephrotoxicity. Neverthess it should be kept in mind that albumin binding impacts transport *in vitro* and thus must be considered when thinking about the clearance of OTA *in vivo* [72]. Thus, a molecular understanding of renal OTA transport will lead to the development of possible strategies for the prevention of OTA-induced nephrotoxicity.
